# Long Non-coding RNA MAFG-AS1 Promotes Cell Proliferation, Migration, and EMT by miR-3196/STRN4 in Drug-Resistant Cells of Liver Cancer

**DOI:** 10.3389/fcell.2021.688603

**Published:** 2021-07-27

**Authors:** Tianming Chen, Bin Huang, Yaozhen Pan

**Affiliations:** ^1^Department of Surgery, Drum Tower Hospital Affiliated to Nanjing University Medical School, Nanjing, China; ^2^The Comprehensive Cancer Center of Nanjing Drum Tower Hospital, The Affiliated Hospital of Nanjing University Medical School & Clinical Cancer Institute of Nanjing University, Nanjing, China; ^3^Department of Hepatobiliary Surgery, The Affiliated Hospital of Guizhou Medical University, Guiyang, China

**Keywords:** long non-coding RNA, MAFG-AS1, miR-3196, STRN4, liver cancer

## Abstract

Long non-coding RNAs (lncRNAs) have been shown to participate in the development and progression of several different types of cancer. Past studies indicated that lncRNA MAFG-antisense 1 (AS1) promotes colorectal cancer. However, the role of MAFG-AS1 in hepatocellular carcinoma (HCC) remains unclear. The aim of the present study is to examine the effect of lncRNA MAFG-AS1 on drug resistance HCC. The results indicated that MAFG-AS1 is upregulated in drug-resistant cells. Further, MAFG-AS1 promotes growth and migration of HCC by upregulating STRN4 through absorbing miR-3196. Thus, LncRNA MAFA-AS1 may become a novel target to treat HCC patients.

## Introduction

Liver cancer is the sixth most ordinary cancer and the third main cause of death of cancer (Altekruse et al., 2014). Liver cancer could be induced by many factors, including hepatitis B virus, alcohol abuse, aflatoxin, and hepatitis C virus infection; non-alcoholic fatty liver disease could also increase the risk of liver cancer specifically (Duan et al., 2014). Although many advances have been achieved in the treatment of liver cancer, the survival rate is still low. Hepatocellular carcinoma (HCC) is proved to be the most common form of liver cancer, taking up 90% of all liver cancers (Feng and Ho, 2014). HCC is featured by rapid growth and invasive tumor and has a tendency for a high probability of metastasis and recurrence (Dika and Abou-Alfa, 2017; Liu et al., 2017). Traditional therapeutic methods for HCC, including surgical resection and chemoradiotherapy, are hard to completely inhibit the tumor growth (Innes et al., 2018). Fundamentally, the molecular mechanisms for HCC metastasis need to be sequentially explored.

Long non-coding RNAs (lncRNAs) are classes of RNAs which are over 200 nt in length without protein-coding ability. LncRNAs can regulate important tumor biological functions, such as epithelial–mesenchymal transition (EMT), invasion-metastasis cascade, proliferation, and drug resistance (Yuan et al., 2014; Li et al., 2016; Tan et al., 2017; Xu et al., 2017). For instance, lncRNA-ATB facilitated cell proliferation in gastric cancer by miR-141-3p/TGFβ2 signaling (Lei et al., 2017). Another study indicated that LncRNA Igf2 was upregulated in HCC cells and tissues and controlled HCC progression through the ERK/MAPK signaling pathway (Bao et al., 2017). Another example is that MAFb ZIP transcription factor G antisense RNA 1 (MAFG-AS1) can expedite cell proliferation and invasion in colorectal cancer by targeting miR-147b/NDUFA4 (Cui et al., 2018). Besides, it is reported that LINC00511 boosted the progression of breast cancer by targeting miR-185-3p/E2F1/Nanog (Lu et al., 2018). MAFG-AS1 is a novel clinical biomarker for clinical progression and unfavorable prognosis in gastric cancer (Li et al., 2020). Downregulation of MAFG-AS1 represses tumorigenesis of colorectal cancer cells through the microRNA-149-3p-dependent inhibition of HOXB8 (Ruan et al., 2020).

However, few studies have provided quantitative evidence of the effects of MAFG-AS1 on HCC progression. Here, we studied the biological functions of MAFG-AS1 in HCC. The impacts of MAFG-AS1 on the regulation of cell invasion, migration, and EMT were also studied. These results provide important evidence that MAFG-AS1 may be a novel therapeutic target and a biomarker for predicting response to sorafenib treatment of HCC.

## Materials and Methods

### HCC Cell Lines

Human HCC cell lines (Huh7, HepG2) and normal liver cell lines (Lo-2) were provided by the Chinese Academy of Sciences Cell Bank (Shanghai, China). In a humidified atmosphere with 5% CO_2_, HCC cells were cultured in Dulbecco’s modified Eagle’s medium (DMEM, Gibco, Grand Island, NY, United States) with 10% fetal bovine serum (FBS, Gibco, Grand Island, NY, United States) at 37°C. The related sorafenib-resistant cell line (HepG2/SF) was generated by exposing cells to increasing concentrations (≤2 μM) of sorafenib.

### Chemicals

The media and antibiotics for cell culture were purchased from HiMedia (Chandigarh, India), Sigma-Aldrich (St. Louis, MO, United States), and Thermo Fisher Scientific China (Shanghai, China). Sorafenib was procured from Santa Cruz Biotechnology, Inc. (Dallas, TX, United States). Sorafenib was dissolved in dimethyl sulfoxide (DMSO) to prepare 1- and 20-mM stocks, respectively, for further use in cell lines.

### Western Blotting

Western blotting was performed according to a standard protocol. Total protein extracts from HCC cells transfected with miR-3196 mimics or miR-3196 inhibitor were loaded on SDS-PAGE gels and transferred into PVDF membranes. After being blocked by skimmed milk, these bands were incubated with primary antibodies at 4°C overnights. Next, membranes were washed with PBS followed by incubation with the appropriate secondary antibody for 1 h at 22–23°C. Finally, enhanced chemiluminescence (ECL, Pierce, Rockford, IL, United States) was added to visualize these membranes. The mouse anti-E cadherin, vimentin, and α-SMA antibodies (R&D Systems Europe Ltd.) were diluted 500 times. Quantification of Western blotting was performed by densitometry using the Storm 820 Phosphor Imager (Molecular Dynamics, Sunnyvale, CA, United States).

### Real-Time Quantitative Polymerase Chain Reaction (qRT-PCR)

qRT-PCR was performed as previously reported (Zhang et al., 2011). Total RNA was extracted with TRIzol. The primers used for gene amplification from the cDNA templates were as follows: α-SMA: 5′-CTGACAGAGGCACCACTGAA-3, 5-CATCTCCAGAGTCCAGCACA-3′; MAFG-AS1: 5′-CGT TCT TAG TTG GTG GAG CG-3′ and reverse, 5′-CCG GAC ATC TAA GGG CAT CA-3′; GAPDH: 5′-AATGG ATTTGGACGCATTGGT-3′, 5′-TTTGCACTGGTACGTGTTG AT-3′. For miRNA, 800 ng RNA was transcribed for cDNA with a reverse transcription kit and miRNA-specific primers supplied by Clontech (Mir-X^TM^ miRNA First-Strand Synthesis, San Francisco Bay, CA, United States). qRT-PCR was performed with miScript SYBR Green PCR Kit, and U6 was used as a normalized control. mouse-miR-3196 mimics, mouse-miR-3196 inhibitors, and their corresponding negative controls were purchased from Qiagen (Dusseldorf, Germany).

### Cell Counting Kit-8 (CCK-8) Assay

Cell proliferation assay was performed according to the instruction of the CCK-8 kit (Solarbio, Beijing, China). Cells at the logarithmic phase were made into single-cell suspension and seeded to 96-well plates with 5 × 10^3^ cells. After seeding, 10 μl of CCK-8 solution mixed with 90 μl of DMEM was added into each well. After 2 h of incubation, absorbance was measured at 450 nm. The 50% growth inhibition (IC50) was measured according to a previous paper (Ma et al., 2017).

### Transwell Invasion and Migration Assay

Transwell assays were performed as described previously (Wang et al., 2017). Briefly, Huh7/R and HepG2/R cells were seeded in Matrigel-coated upper chambers with a pore (50 lL Matrigel, BD Bioscience, United States). Medium without serum and 10% FBS was added into the upper chamber, and medium with 10% FBS was added into the lower chambers. After being incubated for 24 h, the migrated and invaded cells on the lower membrane surface were fixed and then stained with 20% Giemsa solution. Five random fields were counted per chamber by an inverted microscope (Olympus, Japan). Each experiment was repeated three times.

### Wound Healing Assay

Wound healing tests were used to evaluate cell migration in HCCs. Cells were transfected with 80 nmol/l miR-3196 mimics, or 80 nmol/l miR-3196 inhibitors, respectively. Cells are inoculated to produce confluent monolayers in a six-well plate. The fused cells were scraped with a 200-μl sterile pipette. After 48 h of cultivation, the wounds were evaluated using a microscope.

### Immunohistochemistry Staining Analysis

α-Smooth muscle actin (α-SMA) was determined by immunohistochemistry staining. Briefly, sections were dewaxed and endogenous peroxidase activity was quenched by 3% hydrogen peroxide for 15 min. Then, the mixture was blocked with normal goat serum for 30 min to eliminate non-specific binding and incubated overnight at 4°C with primary antibodies against α-SMA (1:100; Abcam). The next day, the specific sections of the sample were treated with a PV6000 Histostain^TM^ kit (ZSGB, Beijing, China) and stained with diaminobenzidine (ZSGB, Beijing, China). Finally, the sections were counterstained by hematoxylin and images acquired with FSX100 (Olympus, Tokyo, Japan).

### Luciferase Reporter Assay

The 3′ untranslated region (UTR) fragments of STRN4 containing the miR-binding sites were amplified by PCR using the cDNA template obtained from the RNA sample of macrophages. The wild-type 3′ UTRs of STRN4 as well as mutant 3′ UTRs with nucleotide substitutions in the putative binding sites corresponding to the seed sequence of miR-3196 were cloned downstream of the firefly luciferase gene in the pGL3 vector (Promega, Madison, WI, United States). Cells were co-transfected with miR-3196 or a control microRNA. Forty-eight hours later, cells were washed in PBS and luciferase activity was measured by a luminometer (Promega, Madison, WI, United States), using a dual-luciferase reporter assay system.

### RNA Immunoprecipitation (RIP)

RIP was performed using the Magna RIP RNA-Binding Protein Immunoprecipitation Kit (Sigma, CA, United States). HepG2 was transfected with miR-3196-biotin or non-sense control (NC)-biotin. Cells were lysed in RIP lysis buffer. A/G magnetic beads with anti-biotin ligation were used to pull down the miR-3196-biotin immunoprecipitation. After the antibody was recovered by protein beads, qRT-PCR was performed to detect STRN4 and miR-3196 in the precipitates.

### RNA Fluorescence *in situ* Hybridization

Fluorescence *in situ* Hybridization (FISH) assay was conducted, referring to the protocol of the kit from GenePharma (Shanghai, China).

### Statistical Analysis

All data were analyzed using SPSS 17.0 software (IBM, Armonk, NY, United States); every experiment was repeated in triplicate, and the data were presented as mean ± standard error (SE) of the mean. Statistical analysis was performed by Student’s *t*-tests. Differences in more than three groups were determined by one-way analysis of variance (ANOVA) test followed by Tukey’s multiple comparison test. *p* < 0.05 was considered statistically significant.

## Results

### High Expression of MAFG-AS1 Is Found in Drug-Resistant Cells of Liver Cancer

**FIGURE 1:**
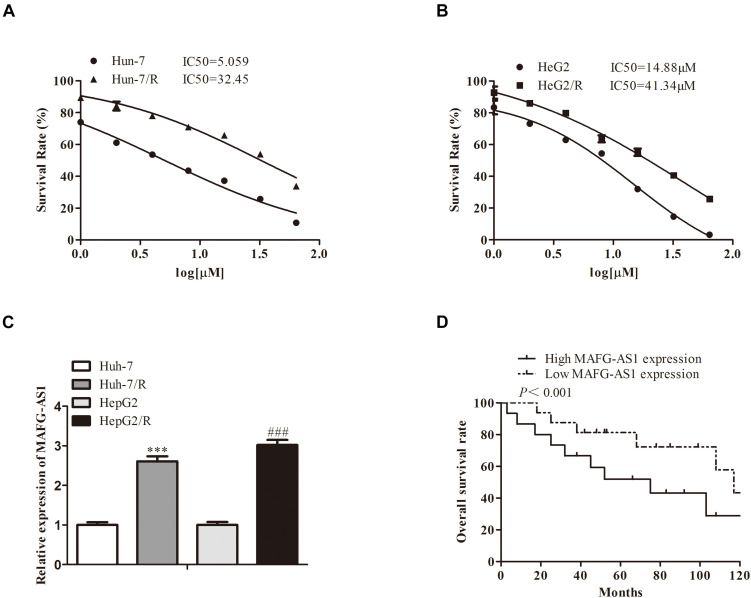
A high expression of MAFG-AS1 is found in drug-resistant cells of liver cancer. **(A,B)** The half-maximal inhibitory concentration (IC50) values of two liver cancer cell lines. **(C)** RT-PCR showed that MAFG-AS1 was upregulated in drug-resistant HCC cell lines (Huh7/R, HepG2/R) compared with parental cell lines. **(D)** The line chart showed the overall survival rate of HCC cell lines with different MAFG-AS1 expression levels.

To investigate the resistance of induced drug-resistant cells, we investigated the cytotoxicity by 3-(4,5-dimethylthiazol-2-yl)-2,5-diphenyl tetrazolium bromide (MTT) assay. After a 48-h treatment with drugs, the growth of HCC cells was markedly inhibited in a concentration-dependent manner. The half-maximal inhibitory concentration (IC50) values of the drug ranged from 5 to 40 μM for two liver cancer cell lines, respectively (Figures 1A,B). Meanwhile, expressions of MAFG-AS1 in drug-resistant cell lines HepG2/R and Huh7/R and parent strain cells HepG2 and Huh7 were detected by RT-qPCR. The results showed that MAFG-AS1 expression in drug-resistant cell lines was obviously higher than that in normal strain cells (Figure 1C). Additionally, the HCC cells showed a longer overall survival rate with a low MAFG-AS1 expression (Figure 1D).

### MAFG-AS1 Knockdown Inhibits Proliferation, Invasion, Migration, and EMT of HCC Cells

**FIGURE 2:**
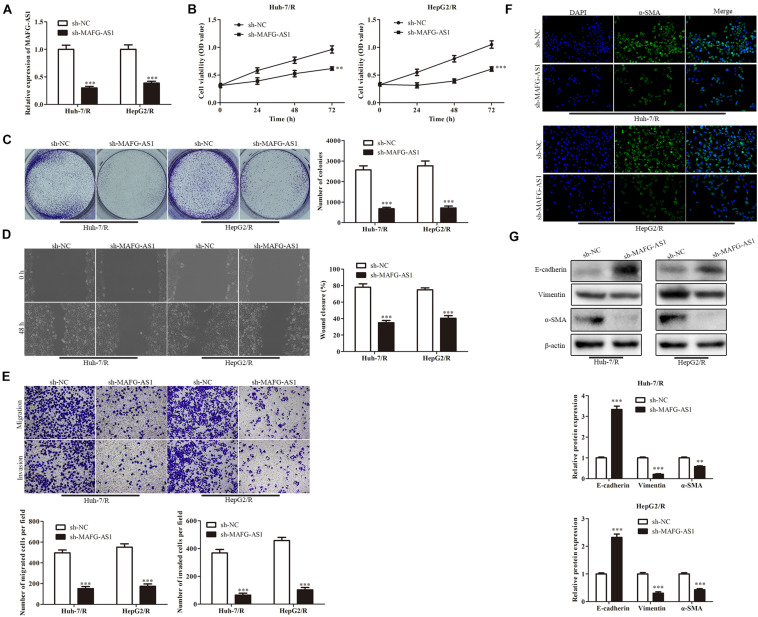
MAFG-AS1 knockdown inhibits proliferation, invasion, migration, and EMT of HCC cells. **(A)** RT-PCR analysis was used to verify the interfering efficiency. **(B)** CCK-8 assay indicated the cell viability in the sh-MAFG-AS1 group compared with the sh-NC group. **(C)** The clone formation abilities of the Huh7/R and HepG2/R cells. **(D,E)** Wound healing assay **(D)** and transwell analysis **(E)** to test the effect on the invasion and migration of HCC cells. **(F,G)** Immunostaining **(F)** and western blotting **(G)** detected the EMT of Huh7/R and HepG2/R cells. Scale bars: 50 μm. **P < 0.01, ***P < 0.001 vs sh-NC group.

To further explore the effect of MAFG-AS1 on drug-resistant liver cancer cell lines, two low-expression MAFG-AS1-resistant cell lines were constructed, and the efficiency was detected by qRT-PCR (Figure 2A). CCK-8 assay was used to detect the viability of cells in each group. The results showed that in contrast to the sh-NC group, the viability of Huh7/R cells in the sh-MAFG-AS1 group was dramatically decreased. Meanwhile, the cell viability was greatly decreased in the sh-MAFG-AS1 group of HepG2/R cell lines (Figure 2B). To further confirm the inhibition of cell proliferation by sh-MAFG-AS1 in liver cancer cells, the colony formation assay and soft agar colony formation assay were conducted on Huh7/R and HepG2/R cells. As shown in Figure 2C, the clone formation abilities of the cells were clearly suppressed by incubation of MAFG-AS1. Next, we performed wound healing analysis to test the effect of MAFG-AS1 on the invasion and migration of HCC cells. The results showed that MAFG-AS1 knockdown significantly reversed liver cancer cell migration (Figure 2D). Moreover, transwell assay showed that in Huh7/R and HepG2/R cells, MAFG-AS1 knockdown decreased the migrated and invasive cell number compared to empty vector-transfected cells (Figure 2E). It has been reported in a previous study that EMT is a critical step of HCC metastasis (Ke et al., 2011). Next, we investigated whether MAFG-AS1 could induce EMT in HCC cells. The results showed that knockdown of MAFG-AS1 could significantly increase the expression of E-cadherin but decrease the expression of vimentin and α-SMA in Huh7/R and HepG2/R cells, as demonstrated by IF or Western blotting (Figures 2F,G). Overall, these results concluded that MAFG-AS1 knockdown inhibited the proliferation, migration, invasion, and tumor growth of drug-resistant HCC cells *in vitro*, suggesting the potential tumor-promoting role of MAFG-AS1 in drug resistance of HCC.

### miR-3196 Bounds With MAFG-AS1

**FIGURE 3:**
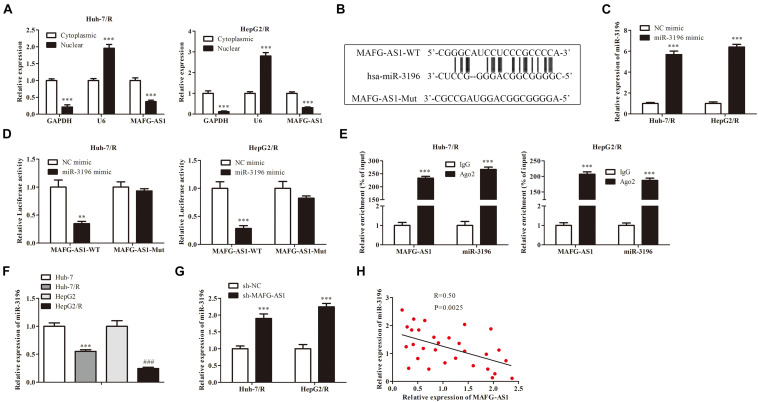
miR-3196 bound with MAFG-AS1. **(A)** RNA-FISH assay implied the location of MAFG-AS1. ***P < 0.001 vs cytoplasmic group. **(B)** Schematic diagram demonstrated the complementarybinding within miR-3196 and MAFG-AS1 3′-UTR with binding sites predicted by bioinformatics programs (StarBase V 2.0, http://starbase.sysu.edu.cn/mirLncRNA). **(C)** RT-PCR analysis was used to verify the efficiency. ***P < 0.001 vs NC-mimic group. **(D)** Luciferase reporter gene assay was performed in Huh7/R and HepG2/R cells transfected with MAFG-AS1 wild/mutant type and miR-3196 mimics/control. **P < 0.01, ***P < 0.001 vs NC-mimic group. **(E)** The relationship between MAFG-AS1 and Ago2 was detected by RIP assay. ***P < 0.001 vs IgG group. **(F)** miR-3196 expression levels were measured in drug-resistant cell lines and parental cell lines using RT-PCR. ***P < 0.001, ^###^*P* < 0.001 vs Corresponding control group. **(G)** RT-PCR showed the miR-3196 expression levels in Huh7/R and HepG2/R cells transfected with MAFG-AS1 shRNA or empty vector. ***P < 0.001 vs sh-NC group. **(H)** Pearson’s correlation analysis of the correlation between miR-3196 and MAFG-AS1.

To explore the mechanism of MAFG-AS1, RNA-FISH assay results were collected and analyzed, which implied that MAFG-AS1 was indeed concentrated in the cytoplasm, indicating that MAFG-AS1 might play a role in the cytoplasm (Figure 3A). To investigate the potential molecular mechanism of MAFG-AS1, bioinformatics prediction tools (StarBase V 2.0)^1^ were used. Results revealed that miR-3196 shared a complementary binding of MAFG-AS1 by binding sites (Figure 3B). To further explore the effect of miR-3196 on drug-resistant liver cancer cell lines, two miR-3196 mimic cell lines were constructed, and the efficiency was detected by qRT-PCR (Figure 3C). Furthermore, luciferase reporter gene assay showed that the luciferase activity was decreased in the combination of MAFG-AS1 wild-type and miR-3196 mimics of Huh7/R and HepG2/R cells (Figure 3D). The relationship between MAFG-AS1 and Ago2 was detected by RIP assay. The results revealed that the specific adsorption level of MAFG-AS1 on Ago2 increased obviously compared to the IgG group of Huh7/R and HepG2/R cell lines (Figure 3E). We also performed RT-PCR analysis for the expression of miR-3196 in Huh7/R and HepG2/R cell lines and parent lines, which indicated that a lower expression was detected in drug-resistant cell lines (Figure 3F). Additionally, the expression of miR-3196 was increased with MAFG-AS1 knockdown (Figure 3G). Afterward, using correlation analysis, we found a negative association between the expressions of miR-3196 and MAFG-AS1 in liver cancer cells (Figure 3H). The above results demonstrate that MAFG-AS1 targets and negatively regulates miR-3196.

### miR-3196 Overexpression Inhibits Proliferation, Invasion, Migration, and EMT of HCC Cells

**FIGURE 4:**
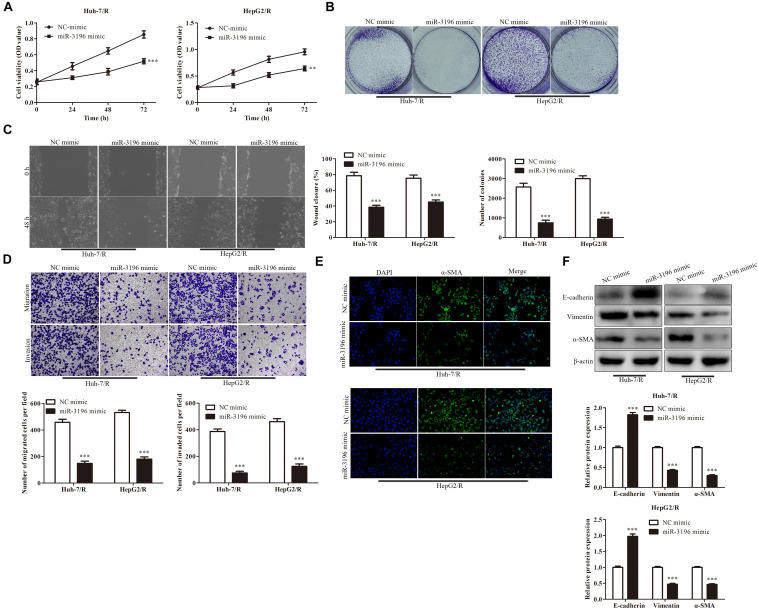
miR-3196 overexpression inhibits proliferation, invasion, migration, and EMT of HCC cells. **(A)** CCK-8 assay indicated the cell viability in Huh7/R and HepG2/R cells transfected with miR-3196 mimic compared to a negative control. **(B)** The colony formation abilities of the Huh7/R and HepG2/R cells in miR-3196 mimic compared to the negative control. **(C,D)** Wound healing assay **(C)** and transwell analysis **(D)** to test the effect on the invasion and migration of HCC cells. **(E,F)** Immunostaining **(E)** and western blotting **(F)** detected the EMT of Huh7/R and HepG2/R cells transfected with miR-3196 mimic compared to a negative control. Scale bars: 50 μm. **P < 0.01, ***P < 0.001 vs NC-mimic group.

We further explore the effect of miR-3196 on drug-resistant liver cancer cell lines. CCK-8 assay was used to detect the viability of cells in each group. The results showed that in contrast to the NC mimic group, the viability of Huh7/R cells in the miR-3196 mimic group was dramatically decreased. Besides, the cell viability was greatly decreased in the miR-3196 mimic group of HepG2/R cell lines (Figure 4A). To further confirm the inhibition of cell proliferation by miR-3196 in liver cancer cells, the colony formation assay and soft agar colony formation assay were conducted on Huh7/R and HeG2/R cells. As shown in Figure 4B, the colony formation abilities of the cells were clearly suppressed by incubation of miR-3196 mimic. Next, we performed wound healing analysis to test the effects of miR-3196 on the invasion and migration of HCC cells. The results showed that miR-3196 mimic significantly reversed liver cancer cell migration (Figure 4C). Transwell assay showed that in Huh7/R and HepG2/R cells, miR-3196 overexpression decreased the invasive cell number compared to empty vector-transfected cells (Figure 4D). Next, we investigated the induction capacity of miR-3196 to EMT in HCC cells. The results showed that overexpression of miR-3196 could significantly increase the expression of E-cadherin but decrease the expression of vimentin and α-SMA in Huh7/R and HepG2/R cells (Figures 4E,F). Overall, results concluded that miR-3196 overexpression inhibited the migration, invasion, and tumor growth of drug-resistant HCC cells, suggesting the potential tumor-promoting role of miR-3196 in drug resistance of HCC.

### MAFG-AS1 Modulates STRN4 Protein Expression Through Targeting miR-3196 in Liver Cancer Cells

**FIGURE 5:**
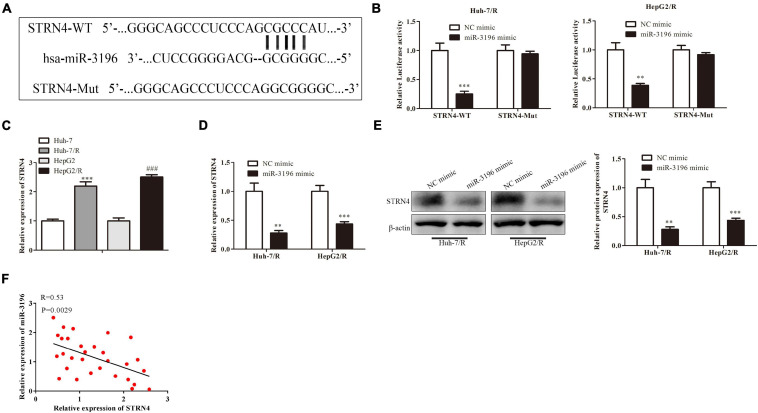
MAFG-AS1 modulates STRN4 protein expression through targeting miR-3196 in liver cancer cells. **(A)** Schematic diagram shows the binding sites within miR-3196 and STRN4 mRNA 3′-UTR. **(B)** Luciferase reporter assay shows the molecular bond within miR-3196 and STRN4 mRNA 3′-UTR. **(C,D)** RT-PCR shows the STRN4 mRNA expression levels in Huh7/R and HepG2/R cells **(C)** or transfected with miR-3196 mimic **(D)**. **(E)** Western blot analysis quantified STRN4 protein expression in Huh7/R and HepG2/R cells transfected with miR-3196 mimic and negative control mimic. **(F)** Correlation analysis found a negative association between the expressions of miR-3196 and STRN4 in liver cancer cells. **P < 0.01, ***P < 0.001, ^###^*P* < 0.001 vs Corresponding control group.

Next, bioinformatics results showed that miR-3196 shared complementary binding sites with STRN4 mRNA 3′-UTR (Figure 5A). Luciferase reporter assay showed that the luciferase activity was decreased when co-transfected with miR-3196 mimics and the STRN4 mild type, suggesting the molecular bond within miR-3196 and STRN4 mRNA 3′-UTR (Figure 5B). We also performed RT-PCR analysis for the expression of STRN4 in Huh7/R and HepG2/R cell lines and parent lines, which indicated a lower expression in drug-resistant cell lines (Figure 5C). In Huh7/R and HepG2/R cells, the STRN4 mRNA expression level decreased when transfected with miR-3196 mimic. Additionally, Western blot revealed that miR-3196 mimic transfection decreased the STRN4 protein expression (Figures 5D,E). Additionally, correlation analysis found a negative association between the expressions of miR-3196 and STRN4 in liver cancer cells (Figure 5F). Overall, results indicated that SRTN4 acted as the target protein of miR-3196, suggesting the regulation of MAFG-AS1 on SRTN4 through targeting miR-3196.

### The Mechanism of MAFG-AS1 Modulating STRN4 Protein Expression Through Targeting miR-3196 in Liver Cancer Cells

**FIGURE 6:**
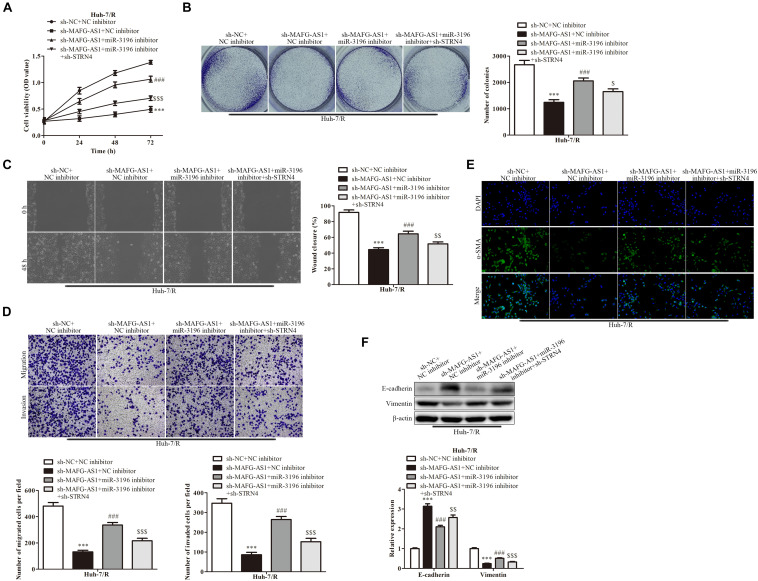
The mechanism for MAFG-AS1 modulating STRN4 protein expression through targeting miR-3196 in liver cancer cells. **(A)** CCK-8 assay indicated the cell viability in Huh7/R and HepG2/R cells transfected with sh-MAFG-AS1, miR-3196 inhibitor, or sh-STRN4 compared to their negative controls. **(B)** The clone formation abilities of the Huh7/R and HepG2/R cells in different groups. **(C)** Wound healing assay **(D)** and transwell analysis to test the effect on the invasion and migration of HCC cells. **(E)** Immunostaining and **(F)** western blotting detected the EMT of Huh7/R and HepG2/R cells transfected with sh-MAFG-AS1, miR-3196 inhibitor, or sh-STRN4 compared to their negative controls. Scale bars: 50 μm. ***P < 0.001 vs sh-NC + NC inhibitor group, ^###^*P* < 0.001 vs sh-MAFG-AS1 + NC inhibitor group, ^$^*P* < 0.05, ^$$^*P* < 0.01, ^$$$^*P* < 0.001, vs sh-MAFG-AS1 + miR-3196 inhibitor group.

In order to investigate whether MAFG-AS1 affects the drug-resistant liver cancer cell lines by regulating the miR-3196/STRN4 pathway, we manipulated the expression of miR-3196 and STRN4 with miR-3196 inhibitor and sh-STRN4, respectively. The knockdown efficiency of miR-3196 inhibitor and STRN4 shRNA-transfected HCC cells was detected by RT-qPCR. Next, CCK-8 analysis showed that sh-STRN4 decreased the activation of Huh7/R cell proliferation induced by a miR-3196 inhibitor (Figure 6A). Meanwhile, colony formation assay analysis showed that the miR-3196 inhibitor reversed Huh7/R cell proliferation by knockdown of MAFG-AS1 (Figure 6B). In addition, wound healing analysis was conducted to test the effect on the invasion and migration of HCC cells. The results showed that the miR-3196 inhibitor significantly reversed liver cancer cell migration, while STRN4 knockdown enhanced this effect (Figure 6C). Next, transwell analysis showed that the migration and invasion ability of Huh7/R and HepG2/R cells increased when these cells were co-transfected with the miR-3196 inhibitor compared with the sh-MAFG-AS1 group. Sh-STRN4 enhanced the reduction in migration and invasion induced by miR-3196 inhibitors (Figure 6D). In addition, our results showed that decreased α-SMA proteins were induced by sh-MAFG-AS1 in Huh7/R cells, while the miR-3196 inhibitor reversed this effect (Figure 6E). The results showed that knockdown of MAFG-AS1 could significantly increase the expression of E-cadherin but decrease the expression of vimentin and α-SMA in Huh7/R and HepG2/R cells as demonstrated by IF or western blotting (Figure 6F). Based on the results, we concluded that MAFG-AS1 regulates the progression of liver cancer cells through the miR-3196/STRN4 axis.

## Discussion

Numerous lncRNAs are aberrantly expressed in HCC, regulating various miRNAs and genes and modulating a variety of biological processes (Chen et al., 2016; She et al., 2016; Han et al., 2017). Therefore, one or more of these lncRNAs may serve as a potential therapeutic target for treating patients with HCC. In the present study, the functional effects and potential underlying mechanism of lncRNA MAFG-AS1 in HCC were examined. Based on the RT-qPCR results, lncRNA MAFG-AS1 was highly expressed in HCC cell lines. After knockdown of lncRNA MAFG-AS1, the proliferation, migration, and invasion of HCC cell lines were significantly decreased. Interestingly, lncRNA MAFG-AS1 and miR-3196 were demonstrated to exhibit a reciprocally negative regulatory association with each other. By inhibiting the expression of miR-3196, MAFG-AS1 promoted the proliferation, migration, and invasion of HCC cells.

Zhang et al. (2018) processed a regulatory network analysis of lncRNAs in colorectal cancer and showed that lncRNA MAFG-AS1 was upregulated in this disease. In addition, high-throughput data analysis and *in vitro* experiments confirmed that lncRNA MAFG-AS1 was highly expressed and affects the proliferation of osteosarcoma cells. Similarly, lncRNA MAFG-AS1 expression was also upregulated in HCC cell lines and primarily distributed in the cytoplasm of HCC cells in the present study. After knockdown of lncRNA MAFG-AS1, the proliferation, migration, and invasion of HCC cell lines were significantly inhibited. Therefore, lncRNA MAFG-AS1 may serve a critical role in the pathogenesis of HCC. This study showed similar results.

Previous studies confirmed the influence of MiRNA in tumor development. Wang et al. suggested that miR-183 promoted MDR in HCC cells by regulating the miR-183-IDH2/SOCS6-HIF-1α feedback loop. Both miR-183 knockdown and SOCS6 overexpression sensitized BEL-7402/5-FU cells to 5-FU (Wang et al., 2016). In this study, we found that miR-3196 could bind with MAFG-AS1. MiR-3196 was lowly expressed in HCC cell lines, and the upregulation of miR-3196 repressed HCC cell proliferative and migratory capacities. Moreover, many studies proved that miRNAs can bind to the 3′ UTR of mRNAs to silence these mRNAs (Tomimaru et al., 2010; Wang et al., 2016). In this research, we confirmed that STRN4 was the downstream target of miR-3196 and demonstrated that STRN4 expression was positively regulated by MAFG-AS1 and negatively regulated by miR-3196.

## Conclusion

In conclusion, MAFG-AS1 was upregulated in HCC cell lines and could bind with miR-3196. Our data hinted that MAFG-AS1 regulated HCC cell growth and migration through the miR-3196/STRN4 axis, exposing a potential neo-biomarker for diagnosis or treatment for liver cancer patients. These results provide important evidence that MAFG-AS1 may be a novel therapeutic target and a biomarker for predicting response to sorafenib treatment of HCC.

## Data Availability Statement

The raw data supporting the conclusions of this article will be made available by the authors, without undue reservation, to any qualified researcher.

## Author Contributions

TC conceived and designed the study and data extraction. BH performed the literature search. YP drafted the manuscript. All authors read and approved the final manuscript.

## Conflict of Interest

The authors declare that the research was conducted in the absence of any commercial or financial relationships that could be construed as a potential conflict of interest.

## Publisher’s Note

All claims expressed in this article are solely those of the authors and do not necessarily represent those of their affiliated organizations, or those of the publisher, the editors and the reviewers. Any product that may be evaluated in this article, or claim that may be made by its manufacturer, is not guaranteed or endorsed by the publisher.

## Footnotes

1
http://starbase.sysu.edu.cn/mirLncRNA
